# Bacterial Microbiota from Lab-Reared and Field-Captured *Anopheles darlingi* Midgut and Salivary Gland

**DOI:** 10.3390/microorganisms11051145

**Published:** 2023-04-28

**Authors:** Najara Akira Costa dos Santos, Vanessa Rafaela de Carvalho, Jayme A. Souza-Neto, Diego Peres Alonso, Paulo Eduardo Martins Ribolla, Jansen Fernandes Medeiros, Maisa da Silva Araujo

**Affiliations:** 1 Programa de Pós-Graduação em Biologia Experimental, Departament of Medicine, Fundação Universidade Federal de Rondônia/Fiocruz Rondônia, Porto Velho 76812-245, RO, Brazil; najara.akira@fiocruz.br (N.A.C.d.S.); jansen.medeiro@fiocruz.br (J.F.M.); 2 Plataforma de Produção e Infecção de Vetores da Malária-PIVEM, Laboratório de Entomologia, Fiocruz Rondônia, Porto Velho 76812-245, RO, Brazil; 3 Multiuser Central Laboratory, Department of Bioprocesses and Biotechnology, School of Agricultural Sciences, São Paulo State University (UNESP), Botucatu 18610-034, SP, Brazil; vanessa.carvalho@unesp.br (V.R.d.C.); jsouzaneto@vet.k-state.edu (J.A.S.-N.); 4 Department of Biotecnology (IBTEC–Campus Botucatu), Instituto de Biotecnologia da UNESP, Universidade Estadual Paulista (UNESP), Botucatu 18607-440, SP, Brazil; diego.p.alonso@unesp.br (D.P.A.); p.ribolla@unesp.br (P.E.M.R.); 5 Programa de Pós-Graduação em Conservação e uso de Recursos Naturais–PPGReN, Departament of Biology, Fundação Universidade Federal de Rondônia, Campus José Ribeiro Filho, Porto Velho 76801-059, RO, Brazil; 6 Laboratório de Pesquisa Translacional e Clínica, Centro de Pesquisa em Medicina Tropical, Porto Velho 76812-329, RO, Brazil

**Keywords:** 16S rRNA, Amazon region, bacteria, malaria vector, microbiota, midgut, salivary gland

## Abstract

*Anopheles darlingi* is a major malaria vector in the Amazon region and, like other vectors, harbors a community of microorganisms with which it shares a network of interactions. Here, we describe the diversity and bacterial composition from the midguts and salivary glands of lab-reared and field-captured *An. darlingi* using metagenome sequencing of the 16S rRNA gene. The libraries were built using the amplification of the region V3–V4 16S rRNA gene. The bacterial community from the salivary glands was more diverse and richer than the community from the midguts. However, the salivary glands and midguts only showed dissimilarities in beta diversity between lab-reared mosquitoes. Despite that, intra-variability was observed in the samples. *Acinetobacter* and *Pseudomonas* were dominant in the tissues of lab-reared mosquitoes. Sequences of *Wolbachia* and *Asaia* were both found in the tissue of lab-reared mosquitoes; however, only *Asaia* was found in field-captured *An. darlingi*, but in low abundance. This is the first report on the characterization of microbiota composition from the salivary glands of *An. darlingi* from lab-reared and field-captured individuals. This study can provide invaluable insights for future investigations regarding mosquito development and interaction between mosquito microbiota and *Plasmodium* sp.

## 1. Introduction

Malaria remains a major infectious disease in the Amazon and the *Anopheles darlingi* (Root) is still the main malaria vector in deforested areas of this region [1]. *Anopheles darlingi* has highly anthropophilic behavior, susceptibility to *Plasmodium* species, and has been shown to be widely adaptable in different areas that highlight its vectorial capacity to transmit malaria parasites [2,3,4]. Nevertheless, this vector is not an isolated entity that interacts only with *Plasmodium* species. Similar to all metazoan, *An. darlingi* harbors a community of microorganisms with which it shares a network of interactions that are important to mosquito development [5]. This community of microorganisms, called microbiota, encompasses bacteria, fungi, viruses, and other organisms [6]. However, the bacterial community has been the most characterized and its role in mosquito biology has been widely examined [7,8].

The bacterial microbiota play roles in the mosquito’s physiology, in addition to being influenced by the mosquito’s life cycle. Mosquito–microbiota interactions range from the larval stage to the adult development stage [9,10,11]; they are involved in blood digestion and fertility [12,13], nutritional support and metabolic process [14,15], mating [16], resistance to insecticides [17,18,19,20], and inhibiting pathogen development [15,21,22]. Additionally, mosquito microbiota bacterial composition is strongly affected by geographical location and seasonal patterns [23,24], mosquito species [25], the developmental stage, and feeding status of the mosquitoes [23,26].

Hence, the characterization of bacterial microbiota remains an important step to understand the microbial ecosystem [27,28] and mainly to identify symbiont bacteria that can be used against *Plasmodium* development inside the mosquito and play a major role in the anophelines’ lifespans. The bacterial microbiota of the salivary glands and midguts of anopheline mosquitoes interact with *Plasmodium* developmental stages, making their characterization critical. While both tissues are key partners in the digestive system, there are more studies on the characterization of the microbiota of the midgut of *Anopheles* compared to the salivary glands of *Anopheles* [29,30,31,32]. Furthermore, it is still unclear which tissue harbors a more diverse bacterial microbiota [29]. However, the age and feeding condition of the mosquito may influence this pattern [30,31,32].

The present study aims to characterize the microbiota composition and diversity from the midgut and salivary glands of *An. darlingi* mosquitoes, the main malaria vector in the Northern region of Brazil. To date, little data on bacterial communities of those malaria vectors are available [33,34,35,36,37]. The study was performed with two different populations of *An. darlingi*. The first one consisted of a lab-reared *An. darlingi* population, which was established as a study model on the development of strategies for malaria control in the Western Brazilian Amazon region [38]; and the second one consisted of field-captured individuals.

## 2. Materials and Methods

### 2.1. Ethics Statement

The study was performed with the permission of the appropriate ethics committee. The research activities are licensed by the Brazilian government, SISBIO No. 65725-1 and SisGen No. A948A47. Ethical approval for protected human landing catches (PHLC) was obtained from the Research Center of Tropical Medicine–CEPEM, under No. 3103187, and the approval of Ethics in Animals Utilization Committees–CEUA 2019/10 of Fiocruz Rondônia for the use of rabbits in colony maintenance was granted.

### 2.2. Sample Collection

To characterize the microbiota of *An. darlingi*, we used two different populations, one from a well-established lab-reared mosquito colony and the other one consisting of a field-captured population (details below).

We used female adult mosquitoes that were three-to-five days old and from the F12 generation. These mosquitoes were exclusively fed on a 15% honey solution from their emergence as pupae until they were dissected for analysis. The mosquitoes were obtained from the lab-reared *An. darlingi* mosquitoes that were maintained at the Production and Infection of Malaria Vector Platform-PIVEM, Fiocruz Rondônia [38]. The lab-reared *An. darlingi* is maintained under controlled conditions at a temperature of 26 °C ± 1 °C, a light cycle of 12 h:12 h (light:dark), a 70% ± 10% relative humidity, and feeding with a 15% honey solution ad libitum. For mosquito production, females are blood-fed on rabbits weekly. Larvae are reared in plastic pans with 1 L of distillate water and fed daily with TetraMin^®^ Marine fish food grounded into sizes of 225 µm, 125 µm, and 63 µm according to larvae stage.

The samples of the *An. darlingi* population from the field were captured using PHLC. The capture was performed at a site located (8°39′8, 874″ S 63°56′8, 106″ W) at Porto Velho, in the state of Rondônia. This location was chosen because the parental mosquitoes of the colony were captured there. The field-captured mosquitoes were stored in plastic cages that were previously cleaned with 70% ethanol and were kept under UV light for 20 min. After the capture, the mosquitoes were transported to our laboratory for mosquito identification according to Consoli and Lourenço-de-Oliveira’s [39] identification keys and organ dissections. Field-captured mosquitoes received no feeding (sugar feeding or blood feeding).

### 2.3. Dissection and DNA Extraction

From each *An. darlingi* group, lab-reared and field-captured, we dissected the salivary glands and the midgut. The midguts and salivary glands were dissected from different mosquitoes to avoid cross contamination during the dissection. Therefore, a total of 60 mosquitoes from each group were dissected. Among them, 30 mosquitoes had their midgut dissected and another 30 mosquitoes had their salivary glands dissected. Before the dissection, the mosquitoes and laboratory equipment were sterilized with 70% ethanol. Mosquitoes were first anesthetized on ice and their surfaces were immediately sterilized with 70% ethanol for five minutes. Afterward, they were rinsed with sterile PBS solution (1X) [28]. The dissection was performed on sterilized slides on a drop of autoclaved 1X PBS using autoclaved needles. Each organ was individually stored in an ATL Buffer (Qiagen, Hilden, Germany) at −20 °C until DNA extraction. Under aseptic conditions, the tissue was ground with a sterile pestle in an ATL Buffer, and the homogenate was followed until whole DNA extraction using the Qiagen DNeasy^®^ Tissue and Blood kit (Qiagen, Hilden, Germany). The DNA extract was eluted in 50 µL of sterile water (SIGMA) and stored at −20 °C until analysis.

### 2.4. Library Preparation and Sequencing

Libraries from each tissue, midgut, and salivary gland were built from a PCR amplification of hypervariable regions of the 16S rRNA. For lab-reared mosquito samples, we amplified the V4 16S rRNA region using region-specific primers (F515: 5′-GTGYCAGCMGCCGCGGTAA-3′; R806: 5′-GGACTACNVGGGTWTCTAAT-3′) that included the Illumina flowcell adapter sequences according to [40]. The reactions were performed with a final volume of 25 µL containing a 1X Taq Platinum PCR Buffer, 3 mM MgCl2, 0.2 µM dNTP, 0.2 µM of the forward and reverse primers, 2.5 U Platinum™ Taq DNA Polymerase (Thermo Fisher, Vilnius, Lithuania), ultrapure water, and 10 µL of genomic DNA. The thermocycling conditions were 94 °C for 3 min followed by 40 cycles of 94 °C for 45 s, 60 °C for 1 min, 72 °C for 1 min and 30 s, and a final extension of 72 °C for 10 min.

For the field-captured mosquito, we amplified the V3–V4 16S rRNA regions using the primers pair, 341F (5′-CCTACGGGNGGCWGCAG-3′) and 785R (5′-GACTACHVGGGTATCTAATCC-3′) [41]. The PCR was performed with a final volume of 25 µL containing 1X GoTaq^®^ Master Mix, 0.4 µM of each primer, ultrapure water, and 10 µL of genomic DNA. Reaction conditions were as follows: 95 °C for 3 min to denature the DNA, with 14 cycles of amplifications at 95 °C for 30 s, 55 °C for 30 s, 72 °C for 30 s, and a final extension of 72 °C for 5 min. We added the adapter sequences of the Nextera XT Index primers (Illumina^®^, San Diego, CA, USA) according to the manufacturer’s protocol. Negative controls were included in all PCRs, and the PCR products were visualized on a 1% agarose gel stained with Gelred^®^.

The libraries were purified using AMPure XP beads (Beckman Coulter, Indianapolis, IN, USA), quantified, and normalized using the NEBNext^®^ Library Quant kit for Illumina following the manufacturer’s protocol. The final pooled libraries of field-captured mosquitoes were adjusted to 6 pM and the pooled libraries of lab-reared mosquitos were adjusted to 10 pM. Sequencing was performed in two runs, one for lab-reared mosquito samples and a second one for field-captured samples using MiSeq Reagent Nano kit v2 (300 cycles) in the Illumina MiSeq Sequencer (Illumina, San Diego, CA, USA).

### 2.5. Sequence Processing and Microbial Diversity Analysis

We used QIIME2 version 2022.8 to analyze the microbiota sequencing data generated. First, we removed low-quality sequences and chimeras with the DADA2 plugin in QIIME2. We used the 16S SILVA reference and taxonomy database (v.138) for taxonomic classification [42]. For lab-reared mosquito sequences, we used a pre-formatted SILVA 138 515F/804R database from [43] (referent to V4 16S rRNA region) to classify filtered sequences. A filtering step was performed using a QIIME2 taxa filter table to remove mitochondrial, chloroplast, and unclassified sequences at the phylum level. For field-captured mosquito sequences, we used a pre-formatted SILVA v.138 full-length database [43] to build a classifier trained only on the region of the target sequence 341F/785R, a referent for the V3–V4 16S rRNA regions using the QIIME2 classify-sklearn plugin.

Statistical analyses were performed with the phyloseq (v.1.38) package in R ambient (v.4.1.2). Alpha and beta diversities were estimated with a sampling depth of 500 sequences. The Chao1, Shannon, and Inverse Simpson indexes were calculated to describe the alpha diversity of the bacterial community from each tissue (salivary gland or midguts), and a Wilcox Mann–Whitney test was used for comparisons. Beta diversity, which indicates dissimilarity between communities, was estimated using unweighted and weighted UniFrac distances for the dissimilarity between the tissues. Principal coordinates analysis (PCoA) and Permutational multivariate analysis of variance (PERMANOVA) were performed to compare intergroup distances. *p*-values < 0.05 were considered statistically significant. ANCOM was performed to analyze the differential abundance between the tissues of each experimental group (lab-reared and field-captured).

## 3. Results

We obtained sequencing data from all sixty individuals analyzed from the lab-reared mosquitoes, which generated a total of 380,983 sequences after MiSeq sequencing of the V4 16S rRNA region. After denoising with DADA2, 93,956 sequences passed in the filtering (a mean of 1,565.9 sequences per sample) (Supplementary Table S1). After filtering sequences associated with mitochondria, chloroplast, and unclassified sequences at the phylum level, a total of 1,041 ASVs (amplicon sequence variants) were obtained from 59 lab-reared mosquito samples.

For field-captured mosquito samples, a total of 307,392 sequences were obtained from MiSeq sequencing of the V3–V4 16S rRNA regions. From these sequences, 134,899 were retained after the denoising step (a mean of 2,248.3 per sample) (Supplementary Table S1). A total of 1,099 ASVs from 45 field-captured mosquito samples were obtained before taxonomic filtering.

### 3.1. Diversity of the Bacterial Community from Midgut and Salivary Gland of Lab-Reared Anopheles darlingi

To account for differences in the sequencing depth of each sample, we rarified data to a depth of 500 sequences per sample to perform alpha and beta diversity analysis. The specified sampling depth retained 21,228 sequences (22.9%) in 43 samples (71.6%) (Supplementary Figure S1).

**Figure 1:**
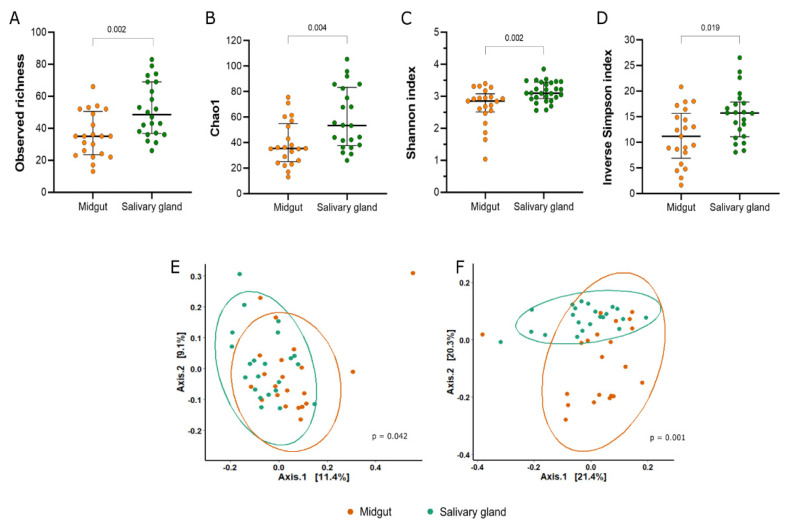
Alpha and beta diversity of bacterial communities from the midgut and salivary glands of colonized *Anopheles darlingi*. The panel shows the (**A**) observed richness, (**B**) estimated richness by Chao 1, (**C**) diversity by Shannon index, and (**D**) inverse of Simpson index. Each point represents an analyzed sample. The bars show the median and 1st and 3rd quartile. The PCoA plots the distances between the samples estimated by unweighted Unifrac (**E**) and weighted Unifrac (**F**).

In general, we observed more ASVs in the bacterial microbiota of the salivary gland samples than in the midgut samples (Mann–Whitney U = 106; *p* = 0.002) (Figure 1A, Supplementary Table S2). The estimation of the Chao1 and Shannon indexes showed that the alpha diversity of the bacterial community sequenced from the salivary gland was significantly different from the midgut microbiota (Chao1: Mann–Whitney U = 115.5, *p* = 0.004; Shannon: Mann–Whitney U = 100, *p* = 0.002) (Figure 1B,C). Although bacterial communities from both tissues were characterized with low uniformity, the Inverse Simpson index was significantly different between the midgut and salivary gland (Figure 1D).

The beta diversity distances showed significant differences in the clustering of midgut versus salivary gland samples (Figure 1E,F). This analysis indicates that the bacterial composition between the tissues was different (unweighted Unifrac: F = 1.4261, *p* = 0.042; weighted Unifrac: F = 4.6736, *p* = 0.001).

### 3.2. Composition and Structure of Bacterial Communities from Midgut and Salivary Glands of Lab-Reared Anopheles darlingi

In total, the sequences analyzed were classified into 18 phylum, 157 families, and 241 genera. In salivary glands and midgut, 99% of bacterial communities were composed of Proteobacteria, Bacteroidota, Actinobacteria, Firmicutes, and Chloroflexi phylum. Other phyla were found in both tissues, although in lower relative abundances (less than 1%) (Supplementary Table S3).

**Figure 2:**
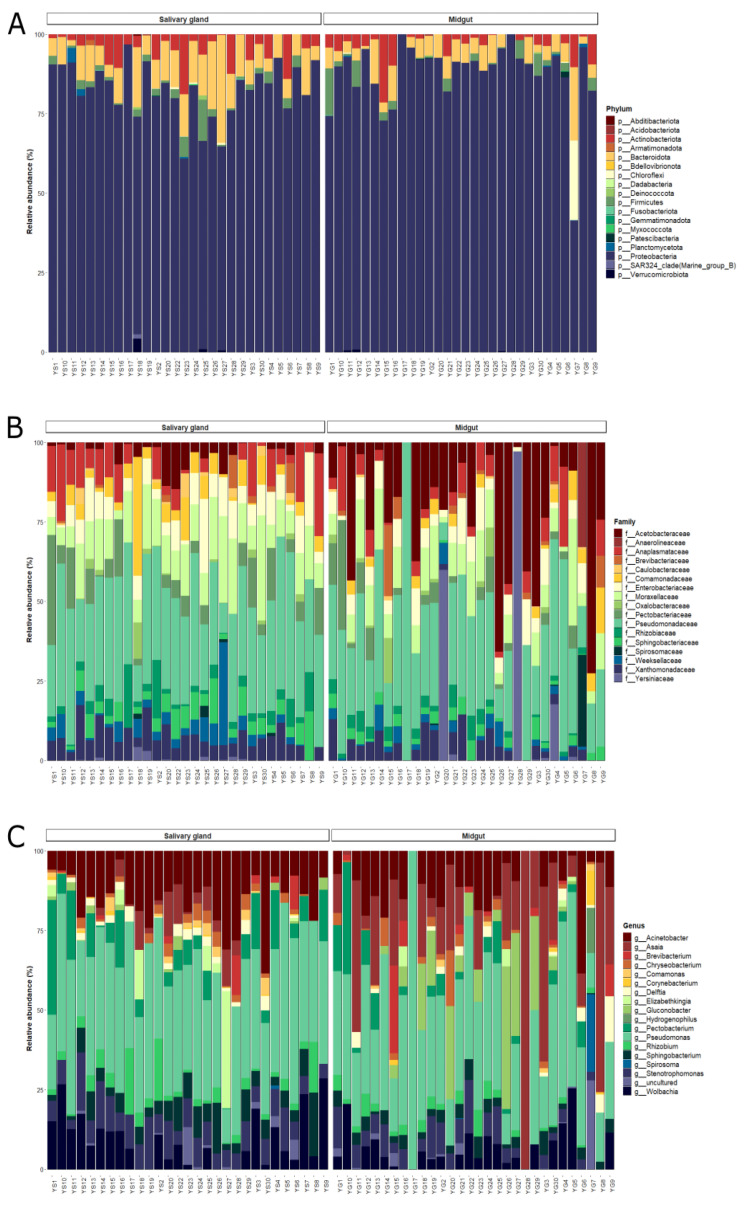
Bar plot of the relative abundances of the bacterial communities in the midgut and salivary gland of lab-reared *Anopheles darlingi* in the taxonomic levels. (**A**) Phylum, (**B**) family, and (**C**) genus.

The Proteobacteria dominated bacterial communities from both tissues, with more than 80% of the relative abundance. The dominance of Proteobacteria was also observed at the family level, with Pseudomonadaceae, Moraxellaceae, Acetobacteraceae, Enterobacteriaceae, Xanthomonadaceae, Yersiniaceae, Pectobacteriaceae, Comamonadaceae, Rhizobiaceae, and Caulobacteraceae composing the group of taxa with more than 1% of the relative abundance (Figure 2A,B, Table S4). Likewise, the more abundant genus, *Pseudomonas*, belonged to Proteobacteria phylum (Figure 2C). Bacteroidetes phylum was more abundant on the salivary gland (12%) compared to the midgut samples (7.1%) of lab-reared *An. darlingi*, while Chloroflexi showed a higher abundance on midguts (3.2%) compared to salivary glands (0.08%). Actinobacteriota and Firmicutes had similar abundances in both bacterial communities (Figure 2A).

In the ANCOM analysis, we found that only the family Comamonadaceae was significantly more abundant on salivary glands compared to midgut samples (W = 51). However, ANCOM analysis did not show significant differences at a genus level between organs. When we use an ASV table, we observe an ASV, identified as *Asaia* sp., more abundantly in midgut samples (W = 1039) (Supplementary Table S6).

Midgut and salivary gland communities shared 32% (76/241) of all genera classified in the taxonomic analysis. The salivary gland showed a greater proportion of unique genus (44%; 105/241), while 60 (25%) genera were unique in the midgut (Supplementary Figure S2). However, the unique taxa harbored in salivary glands have very low abundance, totaling 4.9% of the overall bacterial community. The unique taxon in the midgut totals 8.1% of the overall bacterial community; only two genera exclusive to the midgut showed more than 1% of abundance: the uncultured bacteria of the Anaerolineaceae family (3.6%) and *Hydrogenophilus* (1.8%).

When we looked for a microbiota core at a genus level, we observed that five genera were widely present in both tissues: *Pseudomonas*, *Acinetobacter*, *Sphingobacterium*, *Stenotrophomonas*, and *Wolbachia* (Table 1). Some genera were more prevalent in only one tissue, which was the case for *Asaia*, present in 80% of the midgut samples; *Rhizobium*, *Chryseobacterium*, and *Pectobacterium* were widely distributed exclusively in the salivary gland samples (Table 1).

Other important bacteria commonly found in *Anopheles* mosquitoes were *Serratia*, *Thorsellia*, *Pantoea*, and *Elizabethkingia*, but in low abundances and with a prevalence lower than 50% (Supplementary Table S5). 

### 3.3. Diversity of the Bacterial Community from Midgut and Salivary Glands of Field-Captured Anopheles darlingi

To perform alpha and beta diversity analysis, the rarefaction was performed with a depth of 500 sequences per sample. The rarefaction retained 20 samples (43.4%) and a total of 10,000 sequences (10.9%). Although around 90% of sequences did not pass, the rarefaction curve showed that the diversity plateaued after 500 sequences of depth (Supplementary Figure S3). 

**Figure 3:**
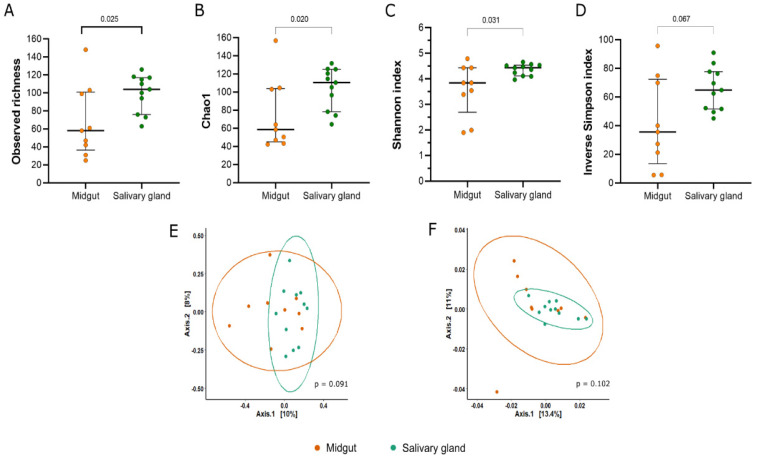
Alpha and beta diversity of bacterial communities from the midgut and salivary gland of field-captured *Anopheles darlingi*. The panel showed the (**A**) observed richness, (**B**) estimated richness by Chao 1, (**C**) diversity by Shannon index, and (**D**) inverse of Simpson index. Each point represents an analyzed sample. The bars show the median and 1st and 3rd quartile. The PCoA plots the distances between the samples estimated by unweighted Unifrac (**E**) and weighted Unifrac (**F**).

The salivary glands showed a higher richness in relation to the midgut, as seen in the observed richness and Chao1 index (observed richness: Mann–Whitney U = 20, *p* = 0.025; Chao1: Mann–Whitney U = 19, *p* = 0.020) (Figure 3A,B). Based on the Shannon index, we observed that the bacterial community from the salivary glands was significantly more diverse compared to the midgut samples (Mann–Whitney U = 21, *p* = 0.031) (Figure 3C). Additionally, both communities showed high dominance (Figure 3D).

In relation to beta diversity, the bacterial communities from both tissues were not statistically different in their compositions according to the unweighted Unifrac (*p* = 0.091) and weighted Unifrac (*p* = 0.102) metrics (Figure 3E,F).

### 3.4. Composition and Structure of Bacterial Communities from Midgut and Salivary Gland of Field-Captured Anopheles darlingi

In general, the ASVs obtained from field-captured *An. darlingi* were classified into 18 phyla, 193 families, and 273 genera. Around 96.3% of the ASVs were classified as Firmicutes, Proteobacteria, Actinobacteria, Bacteroidota, Patescibacteria, and Verrucomicrobiota, with the rest of the ASVs (3.6%) belonging to other taxa (Supplementary Table S7).

**Figure 4:**
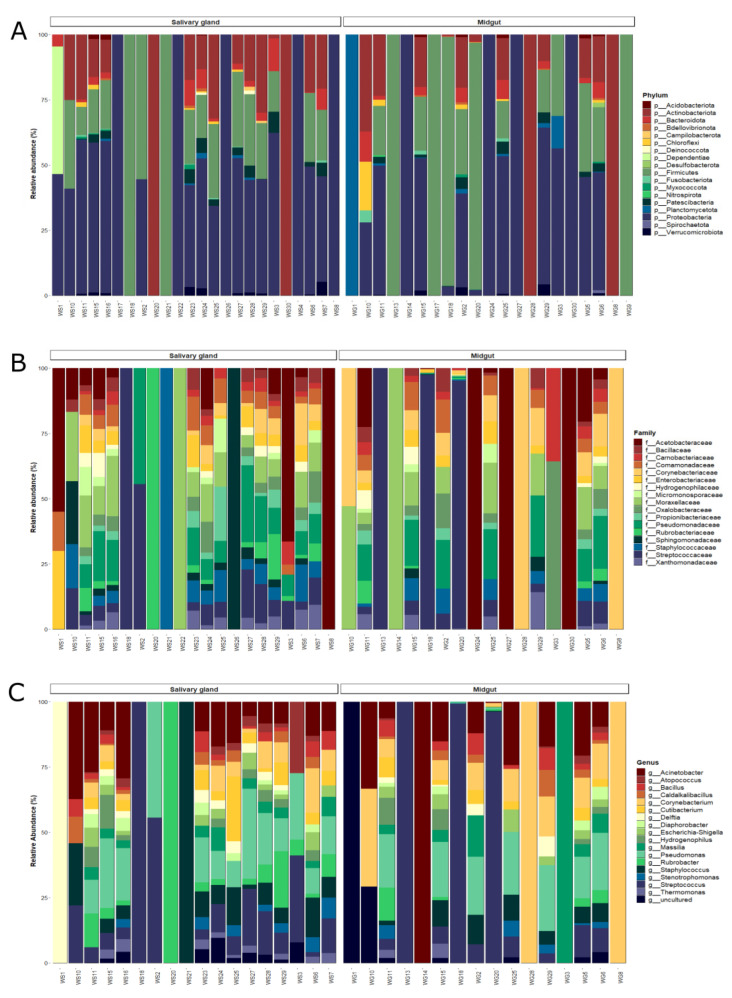
Bar plot of the relative abundances of the bacterial communities from the midgut and salivary gland of field-captured *Anopheles darlingi* in the taxonomic levels. (**A**) Phylum, (**B**) family, and (**C**) genus.

Among bacterial communities of field-captured mosquitoes, some taxa showed an inverse proportion of abundance between the tissues. In the midgut, the Firmicutes had 58.5% of the relative abundance and Proteobacteria had 25.2%, while Proteobacteria was the most abundant in the salivary glands (48.8%) and Firmicutes achieved 20.9% of the relative abundance (Supplementary Table S7; Figure 4A). Actinobacteria showed different distributions between the tissues. In the midgut, Actinobacteria had a relative abundance of 9.8%, and in the salivary glands the abundance increased to 19% (Figure 4A).

Among Firmicutes, the Streptococcaceae family was the most abundant, with the *Streptococcus* genus being the main representative (55.3% of the relative abundance) in the midgut. However, only three samples had *Streptococcus* with more than 90% of the relative abundance, while the abundance ranged from 2.4% to 7.2% in the other samples. *Pseudomonas* from Pseudomonadaceae and *Acinetobacter* from Moraxellaceae were the most abundant genera and families in the salivary glands (Figure 4B, C; Supplementary Tables S8 and S9). Despite that, ANCOM did not find significant differences in taxonomic levels between the tissues.

At the genus level, 36% of the genera (98) were shared between the midgut and salivary glands, 42% (115) were unique to salivary glands, and 22% (60) were only recorded in midgut samples (Supplementary Figure S4). The unique taxa observed in the salivary glands totals 13.8% of the overall bacterial community. Some unique taxa present in the salivary glands were *Asaia*, with 0.7% of the relative abundance, *Neisseria* (0.4%), *Enhydrobacter* (0.4%), *Sphigomonas* (0.4%), and *Shimwellia* (0.3%). In the midgut, the unique taxa composed 5.2% of the entire community.

Observations on the microbiota core of the *An. darlingi* midgut from the field showed that the predetermined prevalence threshold of ≥70% retained only one genus. Because of this, we reduced the prevalence threshold to observe which genera were shared more in most of the samples. At a threshold of ≥50%, we observed two genera, the *Streptococcus* (prevalence = 55.5%) and *Corynebacterium* (prevalence = 61.1%). In the salivary gland, only one genus was present in ≥70%, *Streptococcus*, with a prevalence of 78.9%. When we reduce the threshold to ≥50%, nine genera were included in the salivary gland core: *Pseudomonas*, *Acinetobacter*; *Staphylococcus*, *Rubrobacter*, *Corynebacterium*, *Massilia*, *Delftia*, *Escherichia-Shigella*, and *Cutibacterium* (Table 2).

## 4. Discussion

The salivary glands and midguts of mosquitoes play an important role in the dynamics of the transmission of several pathogens, such as arbovirus and *Plasmodium*. Although the role of midgut microbiota has been largely documented and studied over the years, the bacterial community of the salivary glands, as well its potential performance on pathogen transmission, remains unexplored [29,31,44,45]. The characterization of the bacterial community structure of mosquitoes is important to understand the microbial ecosystem and the interactions between microbiota and host, as well as its impact on pathogen development and transmission and the malaria parasites *Plasmodium* sp. [26,27,28]. In the present study, we characterized the bacterial community of the salivary glands and midgut of *An. darlingi* mosquitoes reared in laboratory along several generations and from field-captured mosquitoes of the Western Amazon region via PHLC.

In general, we observed that the bacterial microbiota of lab-reared or field-captured *An. darlingi* are characterized by a low diversity, with a high proportion of rare taxa compounds having less than 1% of the abundance and the dominance of a few taxa. This pattern of dominance in bacteria microbiota is common between studies on mosquitoes’ microbiota [25,28,46]. Additionally, we found some differences when comparing the tissues, midgut, and salivary gland for each *An. darlingi* population.

The bacterial community of the salivary glands was shown to be richer and more diverse than in the midguts, regardless of mosquito origin. Some studies have demonstrated that salivary glands had higher diversity than midguts in lab-reared mosquitoes: *Anopheles culicifacies*, *Anopheles arabiensis*, *Anopheles merus*, *Anopheles stephensi* and *Culex quinquefasciatus*, and field-captured mosquitoes: *An. gambiae* [29,31]. Sharma et al. [29] proposed that the high diversity of salivary glands may be attributed to the differential digestive processes, such as the acquisition, ingestion, and digestion of feeding. The midgut is a semi-selective environment for bacterial colonization, where a few taxa may successfully live [23,26]. However, other studies recorded different patterns of diversity between the midguts and salivary glands in other mosquito species [31,32]. As Tchioffo et al. [30] had observed, the bacterial richness was higher in midgut samples at mosquito emergence. However, at day-eight after a blood feed, the salivary glands harbored a more diverse bacterial community than the midgut.

For colony lab-reared mosquito samples, the salivary gland and midgut bacterial communities showed a dissimilarity in their compositions based on phylogenetic distances and abundance (unweighted and weighted Unifrac). This suggests that the tissue may be an important factor for bacterial colonization in our lab-reared *An. darlingi* samples, but the same dissimilarity was not observed in the field-captured mosquitoes. It is important to emphasize that the field-captured mosquitoes reflected an amplitude of physiological conditions, such as lifespan, mating, parity, feeding source, and insecticide resistance that were not controlled for this study, which are known to influence the mosquito microbiota [13,26,30,47].

In our data, we observed little to no overlap between the samples of the same tissues in lab-reared and field-captured samples. Even the more abundant taxa in the bacterial communities were distributed irregularly between the individuals. The inter-individual variability of the bacterial microbiota of mosquitoes may be related to the differences in vector competence between individuals of the same population [27,28]. This hypothesis is supported by the knowledge that some metabolites of bacteria may block *Plasmodium* development directly into the anopheline vector [48,49,50]; however, they may also act indirectly as mediators of immune response [21,51,52,53] and mediate peritrophic matrix formation [52,54,55].

As expected, more samples of lab-reared *An. darlingi* harbored the same genera of bacteria in its tissues, which may reflect the laboratory rearing conditions of these mosquitoes. *Pseudomonas* and *Acinetobacter* were the most prevalent genera in the core microbiota of colonized *An. darlingi*, belonging to Proteobacteria phylum, which was the major phylum of the microbiota from lab-reared mosquitoes. These genera are commonly registered in the core microbiota of other anophelines [29,56,57,58], and transmission block activity by a single specimen of these bacteria was observed against *P. falciparum* in vitro and in vivo [48]. A high abundance of *Pseudomonas* and *Acinetobacter* was previously registered in field-captured *An. darlingi* in studies in Colombia and Brazil [23,33,37]. In our field-captured mosquito samples, *Pseudomonas* and *Acinetobacter* were also found in the midgut and salivary glands. 

It is important to note that within the *An. darlingi* colony, the *Asaia* genus was present in salivary glands and had high prevalence and abundance in midgut samples. For field-captured mosquitoes, *Asaia* was present in only one salivary gland sample; however, it was previously found in the midguts of *An. darlingi* mosquitoes in Brazil [34,35,59]. *Asaia* is a symbiotic bacterium that is widely associated with mosquito species and capable of colonizing different tissues, including midguts and salivary glands [60,61]. *Asaia* could be transmitted into the population via different routes, as horizontal transmission during copulation and feeding, or vertically, via the maternal route [62,63,64]. It is, therefore, possible that the laboratory rearing conditions may have favored the transmission of *Asaia* in our colonized *An. darlingi*.

The *Asaia* bacteria has been studied due to its capacity as a tool for paratransgenesis against *Plasmodium* development [61,65]. As Cappelli et al. [66] showed, *Asaia* increased *An. stephensi* resistance against *P. berghei*. In an investigation regarding the reduction in genome size of the bacterial symbiont *Asaia*, Alonso et al. [59] observed a remarkable difference in genetic similarity of *Asaia* isolates from field-captured *An. darlingi* compared to isolates from other species. In the future, it would be important to investigate *Asaia* association with our colonized *An. darlingi* and its role in *Plasmodium* development, especially regarding *Plasmodium vivax* infections.

In colony mosquito samples, we observed ASVs classified as *Wolbachia* genera with an expressive prevalence between midguts and salivary gland samples. *Wolbachia* are endosymbiotic bacteria widely distributed among insects. In anopheline mosquitoes, *Wolbachia* presence was registered in *Anopheles coustani* in Central Africa by Ayala et al. [67]; however, its presence was associated with nematode infection in mosquitoes. Chrostek and Gerth [68] have suggested an independent investigation of sequences to determine *Wolbachia* natural infections in *An. gambiae*, based on the understanding that *Wolbachia* sequences may be introduced in different ways in mosquitoes. Since *Anopheles* naturally infected with *Wolbachia* is still a controversial issue, our register of *Wolbachia* in colonized *An. darlingi* is not sufficient to conclude that this symbiont is present in our samples [68]. 

Other important bacteria, commonly found in mosquito microbiota, were registered in the midgut and salivary gland samples of colonized *An. darlingi*, such as *Pantoea*, *Elizabethkingia*, *Thorselia*, and *Serratia*. These bacteria were detected in the midgut/feces of larvae and *An. darlingi* adults in field samples [33,36,69]. Except for *Thorselia*, all these bacteria have shown the ability to block *Plasmodium* development as bacterial isolates and showed paratransgenesis potential [48,50,70,71,72,73]. Rocha et al. [35] showed that bacterial transformation (GFP) of *Pantoea* and *Serratia* isolates were successfully detected in all developmental stages of *An. darlingi* after feeding adult females.

For field-collected samples, we registered *Escherichia-Shigella* and *Staphylococcus*. These bacteria were commonly associated with *An. darlingi* breeding-site water that, in turn, might contribute to a suitable habitat for this vector [74]. Oliveira et al. [33] found *Escherichia-Shigella* to be the most predominant bacteria in *An. darlingi* positive for *Plasmodium*. Curiously, both *Escherichia-Shigella* and *Staphylococcus* were found in the midgut and salivary glands of colony samples, with *Escherichia-Shigella* showing a low abundance.

Although bacteria with a good potential to arrest *Plasmodium* development in anopheline mosquitoes have been found in our samples, it is important to clarify their role in the sporogonic cycle of *P. vivax*. Recently, an antibiotic-treated *An. darlingi* failed to increase *P. vivax* prevalence and intensity of infection [75,76]. Additionally, the observation that *P. vivax* could manipulate the anopheline detox system [77] and suppress microbiota/immune responses [78] raises the need for additional studies to understand the dynamics of this tripartite interaction: *P. vivax*–*Anopheles*–microbiota. This is especially the case when Neotropical anophelines need to be used as a study model for the development of strategies to control malaria vivax [79].

The bacterial microbiota of mosquitoes is strongly influenced by the environment, feeding, and life stage of these individuals [23,26]. Mosquitoes reared in a laboratory under a standardized procedure tend to lose microbiota diversity in relation to wild mosquitoes [28], especially due to the usage of dechlorinated water and standard insectary procedures for larval stages maintenance [24]. Therefore, a lower diversity in the microbial community is expected among individuals from the colonized populations [28]. In this study, we observed that the bacterial community of field-captured *An. darlingi* was more diverse than that observed in samples from colonized mosquitos, for both the midgut and salivary gland communities. However, there are limitations in comparing the diversity and composition between our lab-reared and field-captured samples because we used two different hypervariable regions of the 16S rRNA gene to sequence bacterial microbiota, and as mentioned before, field-captured mosquitoes usually display a range of physiological conditions that limit the scope for comparisons.

## 5. Conclusions

In the present work, we describe for the first time the salivary gland microbiota of lab-reared and field-captured *An. darlingi*. A greater proportion of lab-reared mosquitoes shared bacterial genera in comparison to field-captured mosquitoes, probably due to the standardized protocols for colony maintenance. Despite that, the intra-variability observed in microbiota composition was probably due to the irregular distribution of bacteria among the samples, which in turn may impact the vectorial capacity of mosquitoes. Finally, the tissues were important for microbiota composition on lab-reared *An. darlingi*, but no differences were observed in field-captured *An. darlingi* samples. The understanding of microbiota composition and structure of our *An. darlingi* colony may guide future investigations regarding mosquito development, as well as increase the understanding of the interactions between the microbiota–vector–*Plasmodium*.

## Figures and Tables

**Table 1:** Prevalence of the bacterial community (≥50%) of the salivary gland and midgut of lab-reared *Anopheles darlingi*.

	Prevalence (%)	
Genus	Salivary Gland	Midgut
*Acinetobacter*	100	86.6
*Pseudomonas*	100	96.6
*Stenotrophomonas*	96.5	73.3
*Rhizobium*	93.1	66.6
*Sphingobacterium*	89.6	83.3
*Wolbachia*	79.3	70
*Chryseobacterium*	72.4	66.6
*Pectobacterium*	72.4	56.6
*Delftia*	68.9	-
*Staphylococcus*	55.1	-
*Asaia*	-	80
*Gluconobacter*	-	53.3

**Table 2:** Prevalence of the bacterial community (≥50%) of the salivary gland and midgut of field-captured *Anopheles darlingi*.

	Prevalence (%)	
Genus	Salivary Gland	Midgut
*Streptococcus*	78.9	55.5
*Corynebacterium*	57.8	61.1
*Acinetobacter*	63.1	55.5
*Pseudomonas*	68.4	-
*Staphylococcus*	63.1	-
*Rubrobacter*	63.1	-
*Bacillus*	57.8	-
*Massilia*	52.6	-
*Delftia*	52.6	-
*Escherichia-Shigella*	52.6	-
*Cutibacterium*	52.6	-
*Atopococcus*	52.6	-

## Data Availability

The datasets generated in metasequencing during the current study are available in the National Library of Medicine-NCBI (Project: PRJNA958445, Access number: SAMN34300140-SAM34300141 for field samples and Project: PRJNA958386, Access number: SAMN34299958-SAM34299959 for colony samples).

## Supplementary Materials

The following supporting information can be downloaded at: https://www.mdpi.com/article/10.3390/microorganisms11051145/s1, Figure S1: Rarefaction curve of observed features from a colonized *Anopheles darlingi*. Each line represents a sample from the midgut or salivary gland. Figure S2: Venn diagram from salivary and midgut samples of colonized *Anopheles darlingi*. Figure S3: Rarefaction curve of observed features from field-captured *Anopheles darlingi*. Figure S4: Venn diagram from the salivary and midgut samples of field-captured *Anopheles darlingi*. Each line represents a sample from the midgut or salivary gland. Table S1: Sequencing summary from the midgut and salivary gland of lab-reared and field captured *Anopheles darlingi*. Table S2: Estimative of alpha diversity index from bacterial community of the midgut and salivary gland of lab-reared and field-captured *Anopheles darlingi*. Table S3: Relative abundance of Phylum from the midgut and salivary gland of lab-reared *Anopheles darlingi*. Table S4: Relative abundance of Family from the midgut and salivary gland of lab-reared *Anopheles darlingi*. Table S5: Relative abundance of Genus from the midgut and salivary gland of lab-reared *Anopheles darlingi*. Table S6: Percentile abundances of features by tissue estimated via ANCOM. Table S7: Relative abundance of Phylum from the midgut and salivary gland of field-captured *Anopheles darlingi*. Table S8: Relative abundance of Family from the midgut and salivary gland of field-captured *Anopheles darlingi*. Table S9: Relative abundance of Genus from the midgut and salivary gland of field-captured *Anopheles darlingi.*
