# Supplementary material for: Bacterial Microbiota from Lab-Reared and Field-Captured Anopheles darlingi Midgut and Salivary Gland
Source: Microorganisms. 2023 Apr 28;11(5):1145. doi: 10.3390/microorganisms11051145 (PMC10224351; doi:10.3390/microorganisms11051145)
Supplement: Supplementary file 1 [file microorganisms-11-01145-s001.zip › microorganisms-2352569-supplementary-figures.pdf]

Supplementary figures

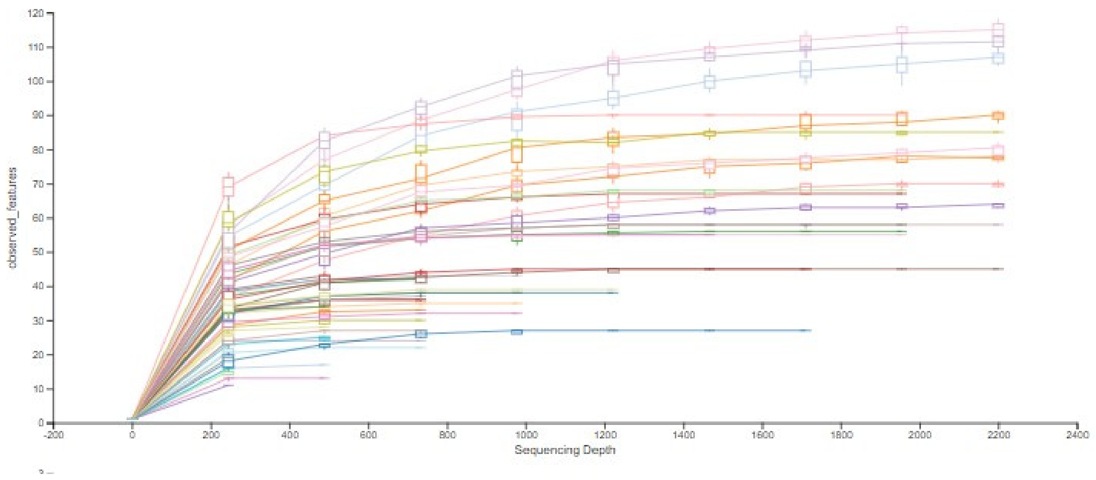

Figure S1 – Rarefaction curve of observed features from colonized *Anopheles darlingi*. Each line represents a sample of midgut or salivary gland.

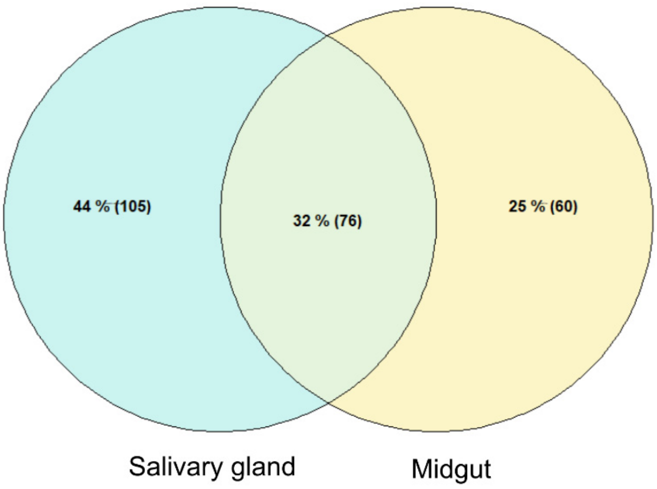

Figure S2 – Venn diagram from salivary and midgut samples of colonized *Anopheles darlingi.*

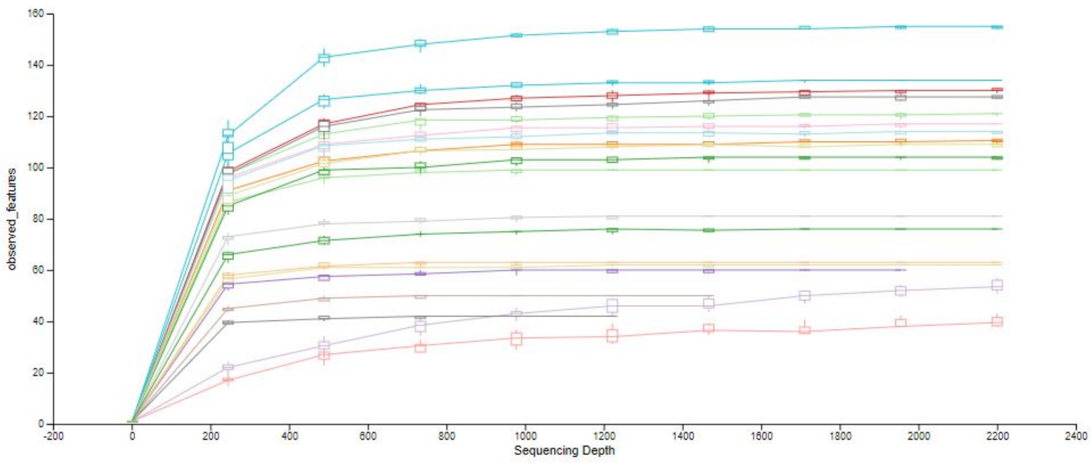

Figure S3 – Rarefaction curve of observed features from field-captured *Anopheles darlingi*. Each line represents a sample of midgut or salivary gland.

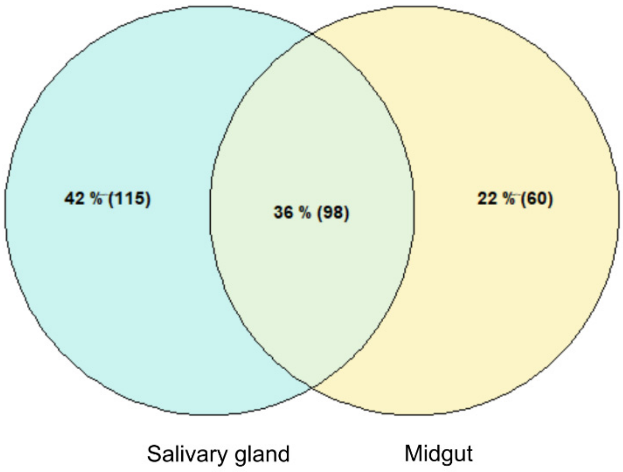

Figure S4 – Venn diagram from salivary and midgut samples of field-captured *Anopheles darlingi.*
